# Influence of Two-Plane Position and Stress on Intensity-Variation-Based Sensors: Towards Shape Sensing in Polymer Optical Fibers

**DOI:** 10.3390/s21237848

**Published:** 2021-11-25

**Authors:** Vitorino Biazi, Letícia Avellar, Anselmo Frizera, Arnaldo Leal-Junior

**Affiliations:** Graduate Program in Electrical Engineering, Federal University of Espírito Santo, Fernando Ferrari Avenue, Vitória 29075-910, Brazil; leticiamunhozavellar.lm@gmail.com (L.A.); frizera@ieee.org (A.F.); leal-junior.arnaldo@ieee.org (A.L.-J.)

**Keywords:** polymer optical fiber, optical fiber sensors, shape reconstruction, multi-plane sensor

## Abstract

Shape reconstruction is growing as an important real-time monitoring strategy for applications that require rigorous control. Polymer optical fiber sensors (POF) have mechanical properties that allow the measurement of large curvatures, making them appropriate for shape sensing. They are also lightweight, compact and chemically stable, meaning they are easy to install and safer in risky environments. This paper presents a sensor system to detect angles in multiple planes using a POF-intensity-variation-based sensor and a procedure to detect the angular position in different planes. Simulations are performed to demonstrate the correlation between the sensor’s mechanical bending response and their optical response. Cyclic flexion experiments are performed at three test frequencies to obtain the sensitivities and the calibration curves of the sensor at different angular positions of the lateral section. A Fast Fourier Transform (FFT) analysis is tested as a method to estimate angular velocities using POF sensors. The experimental results show that the prototype had high repeatability since its sensitivity was similar using different test frequencies at the same lateral section position. The proposed approach proved itself feasible considering that all linear calibration curves presented a coefficient of determination ($R^{2}$) higher than 0.9.

## 1. Introduction

In addition to their traditional use on communication systems, optical fibers are a sensing technology for a series of applications, such as healthcare monitoring [1], environmental changes detection in industries [2] and structural and spacecraft health monitoring [3,4]. This technology has seen substantial growth due to its characteristics and the diversity of parameters that can be monitored, such as temperature, force, acceleration, liquid level, humidity and pressure, among others [5]. In addition, even bacteria [6] and acoustic parameters [7] can be detected with this technology.

These sensors are intrinsically lightweight, small size, chemically stable and with multiplexing capabilities. One of their main characteristics is the immunity to electromagnetic fields, which can distort the expected results from conventional electronic-based sensors [8]. Despite their high signal attenuation, which is inappropriate for long communication systems, polymer optical fibers (POFs) have mechanical properties appropriate for large strain measurements when compared with conventional silica optical fiber. Polymers’ high strain limits and fracture toughness provide mechanical robustness to the stresses applied on POF sensors.

These materials have a lower Young’s modulus when compared with silica optical fiber, which results in more flexible sensors with a higher dynamic range and sensitivity [9]. Additionally, POF sensors also have high biocompatibility and are adequate for biomedical applications, such as monitoring lower limb joint angles during human gait or rehabilitation processes [8]. Regarding wearable applications, POFs are non-brittle and rugged, and therefore they are safer to the user than silica optical fibers, which may puncture the user’s skin when they break [10].

There are different sensor approaches using POFs. Due to their immunity to light source power variations, the ones based on wavelength encoding, such as Fiber Bragg Gratings (FBGs) and interferometers, are important for high precision applications. Nevertheless, their interrogation systems are expensive, heavy and require a large workspace [11]. In contrast, the intensity-variation-based sensors can be portable substitutes to wavelength encoding-based sensors since they use simple and compact electronic components.

This type of POF sensor is an efficient and easy-to-implement alternative when techniques to compensate for its sensitivity to light source deviations are used [12]. The multiplexing capability of the Fiber Bragg Grating (FBG) sensors is a differential characteristic to situations in which it is necessary to measure more than one parameter [13]. However, research has been developed to obtain multiplexed intensity-variation-based sensors that are a less expensive and easier-to-manufacture alternative [14].

Shape sensing consists of techniques used to reconstruct the dynamic 3D shape in real-time for an object by collecting its geometric properties with external equipment without contact or from multiple embedded sensors [15]. This type of analysis may be relevant in the biomedical field for monitoring patients’ posture [16], in maintenance engineering for structural health monitoring [17] and in the aerospace industry to determine the wing shape of composite aircrafts [18] and others. The strategies that use scanners, cameras, and other similar types of equipment have good reconstruction performance; nonetheless, they face some difficulties with occlusion, portability and range of operation [19].

As an alternative, some researchers have been using different types of sensing technologies. An algorithm was developed to reconstruct the surface of a flat polyimide foil under bending that is installed an electrical-based sensor array. This strategy presented an increase in reconstruction errors when a higher bending radius was applied. This happens due to sensor noise at low signal amplitudes caused by electromagnetic interference [20]. A portable solution that can be implemented using smartphones applies 3-axis accelerometers and magnetometers positioned in a grid pattern for real-time shape sensing. In this case, the equipment accuracy also suffers from the effects of electromagnetic perturbations that particularly affect the magnetometers [21].

Optical fiber sensors present a great contribution potential to shape reconstruction technology. The optical fiber characteristics, such as compactness, small size and flexibility, made them useful for these shape sensing applications they need to be attached to the monitored object [15]. Some researchers developed an algorithm to reconstruct the 3D shape of a soft silicone model of an octopus tentacle using FBG sensors and a curve fitting function. The error from this FBG-based process was below to 2.1% showing that this shape sensing strategy is favorably applicable in soft robotics [22].

Another example is a system with FBGs inscribed in multi-core fiber to reconstruct the 3D shape of surgical instruments, such as catheters or endoscopes. The instrument shape is reconstructed applying Frenet–Serret equations in conjunction with curvatures and torsions obtained from sensors embedded on instrument structure [23]. To improve the quality of vascular catheterization treatment, other researchers integrated multiplexed FBG sensors to catheters for 2D/3D spatial reconstruction applying the Helical Extension Method (HEM).

This prototype presented good accuracy with a mean spatial error of 0.41% and repeatability of ±0.82 mm [24]. Although the FBG-based technology is an efficient approach, the inscription of many FBGs on an optical fiber is a difficult and expensive tas,k and compensating the temperature effects in the Bragg wavelength shift is also necessary. Furthermore, a large number of FBGs, grating length and physical separation between them may end up requiring a large sensing area.

This paper presents a polymer optical fiber (POF)-intensity-variation-based sensor with a lateral section for multiplane angle sensing and a methodology to reconstruct the sensor shape. An analytical bending model and simulation of a simply supported POF are presented to show how mechanical stresses and curvatures may influence the sensitivity and optical response of the sensor and to discuss if the proposed approach is feasible for the shape reconstruction process using a POF-intensity-variation-based sensor with a lateral section.

An experimental setup for curvature experiments was used to analyze the sensitivity and linearity of the sensor with the lateral section in four different angular positions at *x*–*z* plane. The tests were performed with flexion and extension angles varying from 0${}^{\circ }$ to 80${}^{\circ }$ and at different frequencies to demonstrate the repeatability of the sensors and the possibility of estimating the angular velocity using a POF-intensity-variation-based sensor.

The proposed strategy uses the sensitivity of the sensor at a constant (or known angular velocity) to estimate its *x*–*z* position and the sensor response after calibration to estimate its *x*-*y* position. The methodology feasibility was evaluated by analyzing the values from the coefficients of determination of the sensor’s calibration curves.

## 2. Theoretical Background

Light conduction through an optical fiber occurs by successive reflections along its length due to the core’s higher refractive index compared with cladding. The fiber core and cladding are surrounded by a jacket that provides robustness and mechanical protection to it. A curvature sensor works under bending, which modifies the fiber geometry and the incidence angle of the light on it. This results in the loss of part of the transmitted light by a refraction to the cladding as the critical angle (for the total internal reflection) changes with the curvature.

The measurement principle of this type of sensor is based on the attenuation of the optical power [12]. If the sensor has a lateral section to improve its sensitivity and linearity, there are more light radiation losses due to the absence of cladding on that region. This mechanism appears in the situation in which the sensitive zone is on the convex side of the curvature. This results in an increase in the number of reflections on the convex side, and then there are more rays escaping than when the fiber is on the straight position [25].

The coupling between the higher and lower guided modes also results in losses by surface scattering [26]. When an optical fiber is mechanically stressed, the stress-optical effect modifies the coefficients of the optical indicatrix, which defines the refractive index of an anisotropic material [27]. Under a bending stress condition, the optical fiber suffers axial and radial variation of its refractive index, which contributes to the optical signal attenuation.

This analysis shows that the direction of the bending, as well as the position of the lateral section in a bending condition for different applied load, affects the stress distribution and length of the sensitive zone, which also affects the signal outputs of the POF curvature sensors.

When a force is perpendicularly applied to the longitudinal axis of an optical fiber that is fixed at its ends, internal bending moments that vary along the fiber longitudinal axis appear. The presence of these internal moments results in stresses and strains on fiber longitudinal direction that cause dimensional and geometric distortions in the optical fiber mechanical structure. The optical fiber size and shape may be altered depending on the external load magnitude and application direction [28]. If the mechanical stresses are insufficient to plastically deform the material, the optical fiber deflection behavior is similar to a slender beam subjected to bending and can be modeled using the theory of elasticity.

Assuming that the material properties are isotropic and the material has linear elastic behavior, Equation (1) is applied to obtain the normal bending stress in a point over a selected cross-section [29]. The points with the same distance to the cross section neutral axis are subjected to the same normal stress.

(1) $$\sigma =\frac{M\cdot y}{I},$$

**Figure 1:**
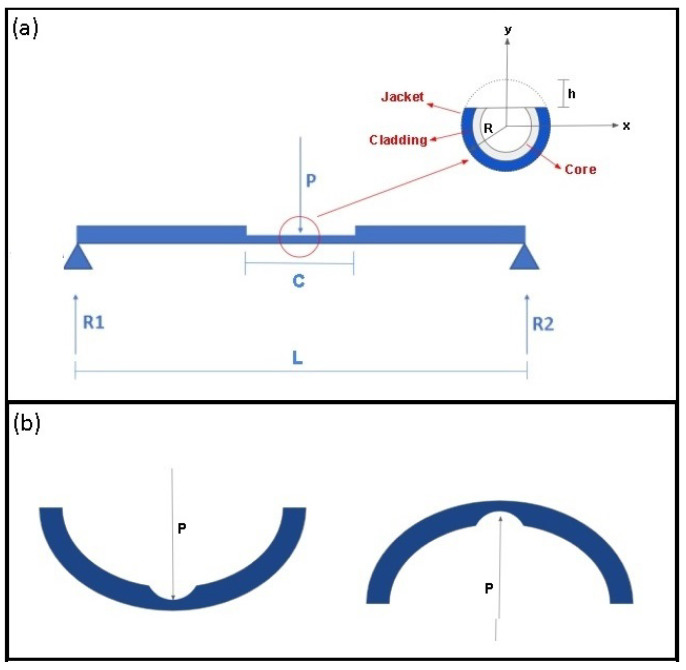
Optical fiber bending configurations. (**a**) Dimensional parameters of a simply supported optical fiber. Figure inset presents the cross-sectional view of the lateral section on the fiber’s top side. (**b**) The bending behavior of optical fiber in different configurations.

*M* is section internal moment, *y* is the distance between the selected point and the section neutral axis and *I* is the section moment of inertia relative to the neutral axis. Figure 1a shows a lateral view of a simply supported optical fiber with a concentrated force *P* applied in the center of the fiber’s longitudinal axis. This configuration may be used to characterize an optical fiber curvature sensor based on signal attenuation. Removing part of the fiber jacket, cladding and core to improve some of the sensor characteristics also have effects in fiber mechanical resistance to bending caused by the change in the cross-section moment of inertia. Figure 1a inset shows a front view of cross-section on the lateral section region.

*R* is the optical fiber radius, and *h* is the depth of removed material. For a bending moment acting on the *x*-axis, the fiber region over the neutral axis is stretched, and the region below the neutral axis is compressed. The position on the plane perpendicular to the fiber longitudinal axis in which the bending force is exerted results in different sensitivities for the curvature sensors. If the perpendicular force is applied on the top side of the fiber as shown in the left side of Figure 1b, the normal stress on the lateral section is compressive and causes a decrease in its length. If the perpendicular force is applied on the opposite side as shown in the right side of Figure 1b, the normal stress is tractive and results in an increase in section length.

The fiber optical characteristics, such as the number of internal reflections inside the sensitive zone (*N*), are dependent on the bending directions [30]. That is caused by the stretch or compression that modifies the section length and affects the optical parameter. In addition, the difference between the compressive and tractive bending stresses results, respectively, in a negative or positive variation on the optical fiber refractive index due to stress optic effect, and this, consequently, affects the attenuation amplitude of the optical signal. For the configuration of Figure 1, the internal moment in the lateral section length may be defined by Equation (2).

(2) $$M\left(x\right)=\{\begin{array}{c} \frac{P\cdot x}{2},\frac{L-C}{2}<x<\frac{L}{2}\\ \frac{P\cdot x}{2}-P\cdot \left(x-\frac{L}{2}\right),\frac{L}{2}<x<\frac{L+C}{2} \end{array},$$

Hooke’s Law is the equation that relates linearly, within the elastic region, the normal stress and normal strain in the same direction that occur in elastic materials under mechanical loading as shown in Equation (3). The proportionality constant is the material’s Young Modulus [29].

(3) $$E=\frac{\sigma }{\epsilon },$$

*E* is the material’s Young Modulus, σ is the normal stress and ϵ is the normal strain. By using the previous equations and the strain definition it is possible to find the fiber length after the bending application. The average normal strain may be defined as the ratio between the length variation (*C*) and the original length ($C_{0}$) of a deformed body [29] as shown in Equation (4).

(4) $$\epsilon =\frac{C-C_{0}}{C_{0}},$$

To demonstrate the dependence between the signal variation rate and the lateral section position on the bending application, an analysis using the equations that define the bending stresses and strains was made for four bending directions. For two cases, in which the force is applied perpendicularly over the lateral section and perpendicularly on the opposite side of the lateral section, the cross-section moment of inertia relative to the *x*-axis on section length is obtained by Equation (5).

(5) $$Ix_{t}=Ix_{b}=2\cdot \int_{-R}^{R-h}y^{2}\cdot \sqrt{R^{2}-y^{2}}dy,$$

where $Ix_{t}$ and $Ix_{b}$ are the cross-section moment of inertia when the inferior portion of the lateral section is parallel to the *x*-axis. Ixt is for the lateral section positioned above the *x*-axis and Ixb is for the lateral section positioned below the *x*-axis. Using the equations from 1 to 5, it is possible to verify the effect of the bending stress on the section length that also has an influence on sensor optical characteristics during the measurements. Equation (6) provides the lateral section final length with load applied on fiber upper region and Equation (7) gives the lateral section final length with load applied on fiber lower region.

(6) $$C=Co-\frac{P\cdot (R-h)}{2\cdot Ixt\cdot E}\cdot \left(\int_{\frac{L-C}{2}}^{\frac{L}{2}}x^{2}dx+\int_{\frac{L}{2}}^{\frac{L+C}{2}}Lx-x^{2}dx\right),$$

(7) $$C=Co+\frac{P\cdot (R-h)}{2\cdot Ixt\cdot E}\cdot \left(\int_{\frac{L-C}{2}}^{\frac{L}{2}}x^{2}dx+\int_{\frac{L}{2}}^{\frac{L+C}{2}}Lx-x^{2}dx\right),$$

By altering the direction in which the load is applied, the moment of inertia relative to the *x*-axis is altered. For the situation in which the inferior portion of the lateral section is perpendicular to the *x*-axis and the applied concentrated force is parallel to the y axis, the moment of inertia relative to the neutral axis on the section portion may be obtained by Equation (8).

(8) $$Ixl=\frac{2}{3}\cdot \int_{-R}^{R-h}R^{2}-y^{2}\cdot \sqrt{R^{2}-y^{2}}dy,$$

For the previous situation, the bending stress varies linearly along lateral section transversal length and the lateral section has a final longitudinal length for each part of its transversal length. Nevertheless, the relation between the force and the length variation remains linear and the transversal length varies from $-\sqrt{h\cdot (2\cdot R-h)}$ to $\sqrt{h\cdot (2\cdot R-h)}$ and is obtained from Equation (9).

(9) $$C=Co+\frac{P\cdot y}{2\cdot Ixl\cdot E}\cdot \left(\int_{\frac{L-C}{2}}^{\frac{L}{2}}x^{2}dx+\int_{\frac{L}{2}}^{\frac{L+C}{2}}Lx-x^{2}dx\right),$$

An analysis may also be performed to show that the maximum fiber deflection is dependent on the load applied on the perpendicular plane- to the fiber axis. The classical Euler-Bernoulli beam theory is not easy to apply for this analysis due to the fiber’s non-uniform cross-section that results in nonlinear differential equations. In order to enable these calculations, an analysis using Castigliano’s method in which the partial derivative of the total strain energy of a structure with respect to a generalized force gives the generalized displacement in the force direction [29].

For the simply supported optical fiber with a centered concentrated load, the total strain energy has contributions of bending and shear energies. Assuming isotropic characteristics and that the material Young’s Modulus and Shear modulus is constant along the fiber axis, Equation (10) provides the maximum fiber deflection.

(10) $$\delta_{c}=\int \frac{M\left(X\right)\cdot \frac{dM(x)}{dP}}{E\cdot I(x)}dx+\int \frac{f_{s}\cdot V\left(X\right)\cdot \frac{dV(x)}{dP}}{G\cdot A(x)}dx,$$

$f_{s}$ is the form factor, specific for each cross-section, V(x) is the internal shear force, *G* is the material shear modulus, and A(x) is the cross-section area. To solve this mathematical model, the optical fiber can be considered as a thin beam, and the shear strain energy is neglected compared to the bending strain energy [29].

The lateral section longitudinal length as a function of the concentrated load was obtained from the mathematical model. Using the Figure 1a inset as a reference and assuming that the load is always on the top side of fiber cross section, there are three cases:

(1) The lateral section on the top side of the fiber cross-section and the load is over it.
(2) The lateral section on the bottom side of the fiber cross-section and the load is on the opposite side.
(3) The lateral section is on the lateral sides of the fiber cross-section positioned perpendicular to the *x*-axis and the load is on the top side of the fiber.

The moment of inertia of the lateral section region relative to the neutral axis and the fiber maximum deflection is the same for the case in which the lateral section is on the top side of the fiber cross-section, and the load is applied over it and for the case in which the lateral section is on the bottom side of the fiber cross-section and the load is applied on the opposite side. The third case presents a distinct moment of inertia relative to the *x*-axis and deflection value. Table 1 shows the optical fiber mechanical parameters used to check the bending mathematical model results. The information was collected from the literature [30,31].

**Figure 2:**
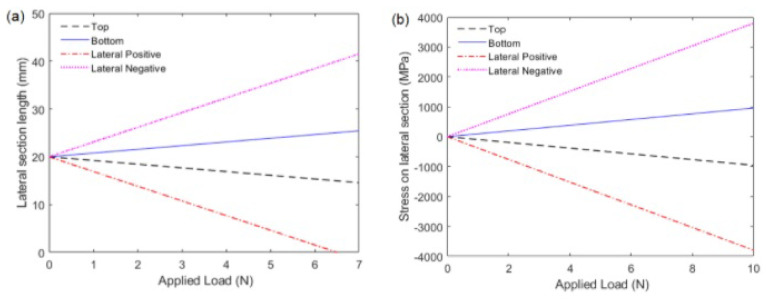
Stress and strain effects on optical fiber under bending. (**a**) Stress on the lateral section positioned on the top, bottom and lateral sides of the fiber as a function of the applied load. (**b**) The final longitudinal length of the lateral section positioned on the top, bottom and lateral sides of the fiber as function of the applied load.

The graphs of Figure 2 show the values of lateral section stress and longitudinal length as a function of the concentrated load for the three aforementioned cases. For the third case, *y* was fixed as the maximum and minimum values for lateral section transversal length. An analysis of the three different approaches enables us to observe that the mechanical stresses and length variations lead to a different optical response for the sensor in each situation. Proper treatment of the sensor data can be used to estimate the curvature radius position and the fiber bending direction by knowing the sensor sensitivity for each cited case. This allows obtaining sensors for applications, such as shape reconstruction using a smaller number of sensing elements and lower-cost systems.

**Figure 3:**
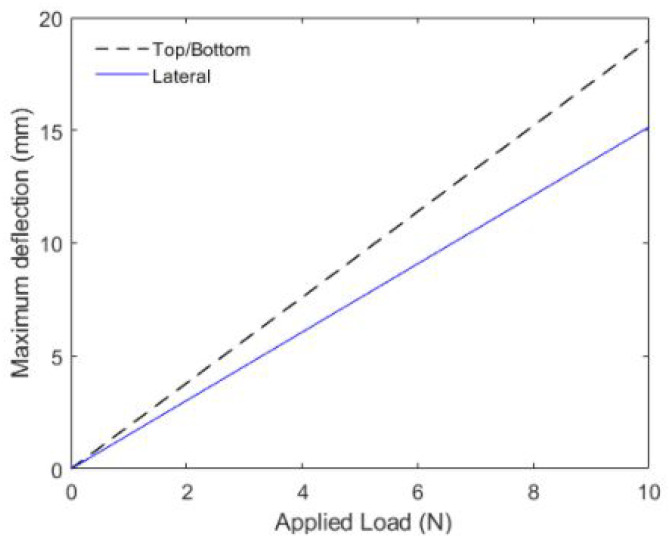
Maximum deflection of the simply supported optical fiber under effect of a load applied on its longitudinal center. The figure shows the values for the lateral section on the top/bottom and lateral side of the fiber.

Figure 3 shows the variations on the fiber curvature as a function of section moment of inertia when the same bending load is acting on the fiber. The influence of the deflection on the fiber optical response is directly related to the stresses and length variation analysis considering that both are physically related. The sensitive zone length also has an influence over the POF sensor response, affecting its hysteresis, sensitivity and linearity. The relation between the lateral section length and these parameters was presented in [32].

Assuming only elastic characteristics for polymer optical fibers leads to modeling errors in the sensor response since the polymers have elastic and viscous characteristics when they are mechanically excited. In this case, the mechanical stresses and strains are time-dependent functions and stress-optic effect modeling has to be performed, such as in [28].

For this reason, viscoelastic modeling is necessary to compensate for the hysteresis effect that appears during cyclic applications [33]. However, since the curvature sensors present high linearity without viscoelastic compensation and their sensitivity has no direct correlation with hysteresis [33], the elasticity theory is an appropriate model to evaluate the experimental analysis from this work. The presented model is based on the linear Bernoulli beam, which is valid only for small displacements where the fiber cross-section remains on the plane within the curvature.

However, it is possible to infer from the graphs that, even for small values of displacement, the direction in which the fiber undergoes curvature would influence the sensitivity of the sensors. If a simulation of larger deformation is required, numerical methods can be used, generally with the finite element method, instead of the analytical method presented here. Nevertheless, the case at which there are larger deformations in the optical fiber is experimentally verified in the next sections.

## 3. Materials and Methods

As the intensity variation curvature sensor operation principle is based on the bending induced loss when the optical fiber is under curvature, the experimental setup for the curvature evaluation at different spatial conditions comprises a servomotor with position and velocity control attached to a potentiometer that provides the position feedback for the control system. The POF used in this work has a PolyMethyl Methacrylate (PMMA) core and cladding of the same perfluorinated material, which results in refractive indices of 1.492 and 1.417 for the core and cladding, respectively.

Regarding the fiber’s dimensions, the POF has a 980 μm core and cladding thickness of 10 μm. A lateral section is performed on the fiber through abrasive removal of material at length and depth of 20 mm and 0.6 mm, respectively, following the guidelines presented in [32]. The manufacturing process of the sensitive zone region is performed by fixing two ends of the POF to mitigate movements during the abrasive removal of material. Then, a rotary machining tool is positioned in movable support with the controlled position in 2D plane (for the control lateral section length and depth).

Thereafter, the tool performs the machining of the POF with controlled parameters (depth and length). It is worth mentioning that the lateral section removal creates a D-shaped POF in which the optical power varies with the angle. Such an operation principle is different when compared with the ones based on microchannel [34], grating [35] or interferometer [36] structures, where there is a wavelength variation as a function of the strain (or stress).

**Figure 4:**
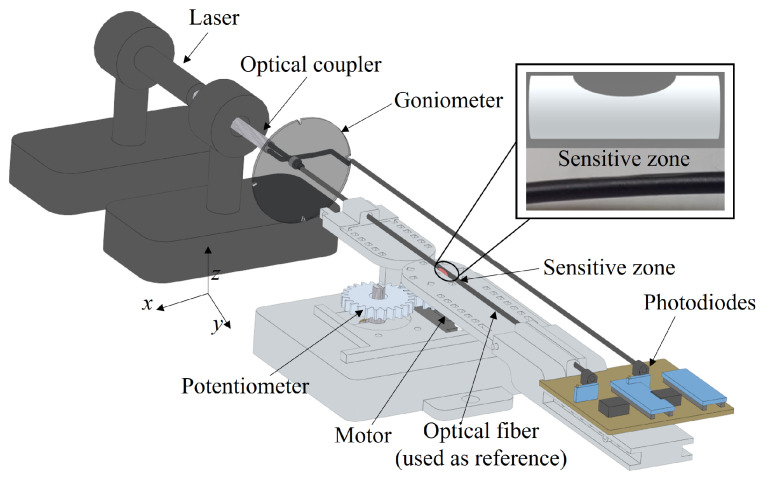
Experimental setup for the multiplane curvature analysis.

Figure 4 shows the experimental setup for the curvature experiments, where there is a fixed part and a movable part, attached to the motor unit in which the curvature at controlled angles and angular velocities occurs at the *x*–*y* plane. The fiber is positioned in the setup with the center of the lateral section aligned with the center of curvature of the setup. Furthermore, a goniometer is positioned at the *x*–*z* plane (see Figure 4) to provide the angular positioning of the lateral section. The tests are performed in a temperature-controlled environment with a temperature of 24 ${}^{\circ }$C ± 1 ${}^{\circ }$C.

The test comprises of flexion and extension cycles (in the *x*–*y* plane) with constant angular velocity and angular range of 0${}^{\circ }$ to 80${}^{\circ }$ in sequential cycles at each lateral section angular position. The angular position of the lateral section is changed by turning the optical fiber at the *x*–*z* plane with the aid of the goniometer. Five flexion/extension cycles are performed with the POF sensitive zone at each angular position at the *x*–*z* plane, namely 0${}^{\circ }$, 90${}^{\circ }$, 180${}^{\circ }$ and 270${}^{\circ }$, where the optical power variation is acquired by a photodiode (IF-D91, Industrial Fiber Optics) with transimpedance amplifier connected to a signal acquisition unit (USB-6008 board, National Instruments) at an acquisition frequency of 200 Hz.

The light source is a laser of 3 mW centered at 650 nm. Therefore, the experimental steps comprise of the lateral section machining via abrasive removal of material. Then, the D-shaped POF is positioned in the experimental setup for angle assessment, at different orientations of the region at which the material was removed. For each condition, the angle is varied (in the aforementioned range) as well as the velocities (referred to as the frequency variations).

The signal analysis is performed by means of comparison between the sensor and reference optical fibers to compensate for possible variations on the light source power, which can lead to measurement errors with intensity-variation-based sensors [32]. The optical power variation is compared with the angle measured by the potentiometer at each position of the goniometer at the *x*–*z* plane. Thus, the angular variation (from 0${}^{\circ }$ to 80${}^{\circ }$) at *x*–*y* plane is analyzed at each position on the *x*–*z* plane, i.e., 0${}^{\circ }$, 90${}^{\circ }$, 180${}^{\circ }$ and 270${}^{\circ }$. In this case, the sensitivities of the sensors for the angle variation of 0${}^{\circ }$ to 80${}^{\circ }$ at each plane are compared and analyzed as a function of the stress distribution shown in Figure 2, where there are different slopes for different lateral section positions.

The sensitivities at different lateral section positions can be analyzed to infer the optical fiber positioning at the *x*–*z* plane, whereas the optical power variation can be used to estimate the angle position at the *x*–*y* plane. It is worth noting that this analysis is performed under constant (or known) angular velocity conditions, which commonly occur in many mechatronic devices and wearable robots for rehabilitation purposes, as shown in [37].

However, in applications where the angular velocity is not constant or not previously known (e.g., wearable sensors for movement analysis), it is possible to acquire the angular velocity using a complementary sensor system or using the reference optical fiber (shown in Figure 4) not only as a reference for light source optical power variation but also for the angular velocity measurement. In order to verify the possibility of measuring the angular velocity with another POF-intensity-variation-based sensor, periodic flexion/extension cycles were performed at three different frequencies namely 0.004, 0.015 and 0.025 Hz, which correspond to three angular velocities.

Such frequencies were chosen following the maximum and minimum frequencies provided by the motor unit for this angle range. To estimate the oscillatory movement frequency, the Fast Fourier Transform (FFT) is applied to the sensor responses. However, it is also possible to obtain the angular velocity from the signal derivative even in non-periodic movements.

After the angular velocity estimation, the sensitivities of the sensors at each lateral section position are analyzed, where the sensitivity value is used to estimate the lateral section position at the *x*–*z* plane. Then, the sensors’ responses are normalized and a linear regression is applied to the sensors’ responses in order to obtain the angular position at the *x*–*y* plane.

## 4. Results and Discussion

**Figure 5:**
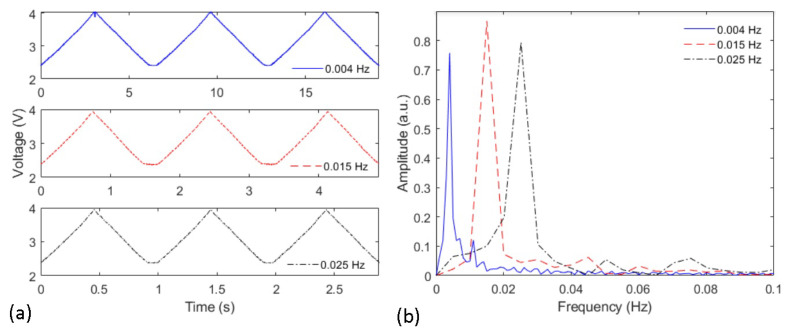
(**a**) Voltage variation as a function of the time for the sensor at each frequency. (**b**) FFT of the sensor response at each angular velocity condition.

The optical power variation in angle cycles (from 0${}^{\circ }$ to 80${}^{\circ }$) at different angular velocities is presented in Figure 5 in sequential cycles as a function of time. It is possible to observe the differences in the time range for all three presented cycles in Figure 5a. It is important to mention that these cycles were performed with the same optical fiber at the same angular position of the lateral section. Considering all angle cycles shown in Figure 5a, there is a variation from 4.0 to 2.4 V in all flexion/extension cycles, which indicates similar sensitivities in all analyzed cases. Thus, the differences between the cycles at each frequency were observed in the time axis, whereas there was no significant sensitivity variation when all sensors responses are compared.

Figure 5b shows the frequency responses of the sensor after applying the FFT at each cycle, where there is a frequency shift when all cases are compared. It is also important to mention that the peak frequency corresponds to the frequency of the oscillatory movement at each condition. In addition, some side lobes on the spectra are related to the vibrations on the motor and on the setup structure. Nevertheless, the sensors’ responses indicated the possibility of using the optical fiber sensor as a tool for frequency (and angular velocity estimation), which is useful on the multiplane and shape reconstruction analysis of the intensity-variation-based POF curvature sensor.

**Figure 6:**
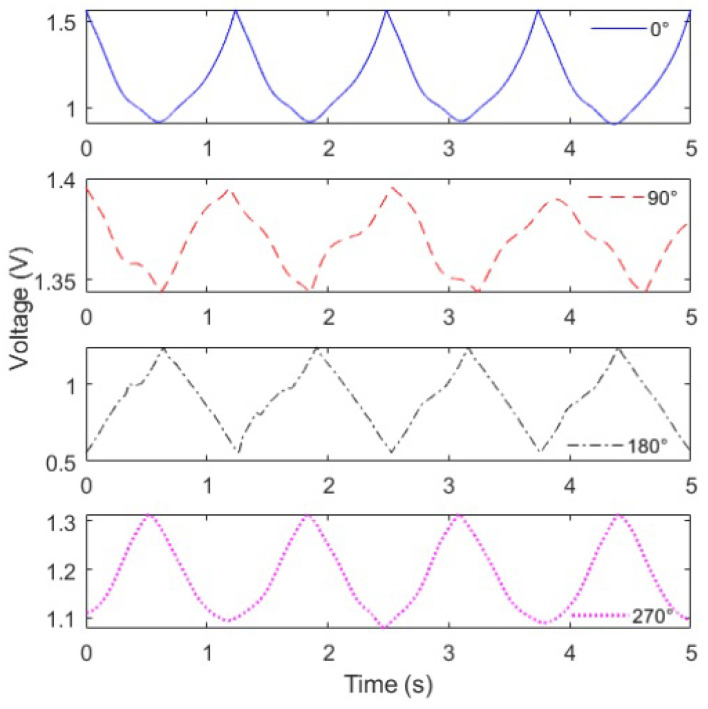
Optical power variation (represented by the voltage variation) of the POF sensor for different positions of the lateral section at flexion and extension cycles (from 0${}^{\circ }$ to 80${}^{\circ }$).

Thereafter, the angular variation was applied on the POF at different lateral section positions. Figure 6 shows the optical power variation (represented as the voltage variation in the output of the transimpedance amplifier) as a function of time for the 0–80${}^{\circ }$ cycles. From the optical power variation at each condition, it is possible to infer differences in the sensor sensitivity as a function of the lateral section angular position at the *x*–*z* plane. Furthermore, these different positions of the lateral section can also lead to differences in the sensor linearity, where we can observe some variations in the shape of the sensors’ responses. Such differences can be related to additional transverse stress components applied on the optical fiber when it is rotated to the desired angle of the lateral section.

However, the coefficient of determination ($R^{2}$) of the sensor’s responses in all lateral sections angular positions is still higher than 0.9, which indicates a high correlation with a linear response. Thus, the angle estimation (at *x*–*y* plane) is performed by means of a linear regression between the normalized optical power variation (represented as the voltage) and the measured angle.

**Figure 7:**
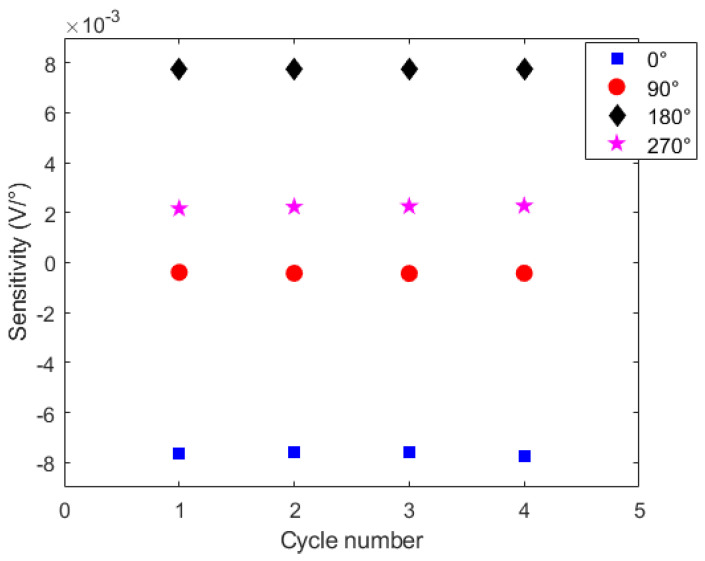
Sensor sensitivities for all cycles at each tested lateral section positioning.

In order to verify the sensitivity at each lateral section position, Figure 7 shows the sensitivity of the sensors (in V/${}^{\circ }$) at each cycle. Compared with the simulations presented in Figure 2, the experimental results of Figure 7 indicate a correlation with the stress calculated for each lateral section position. The slopes of each response of Figure 2, presented a similar pattern of the ones shown in Figure 7, where there is a smaller slope at top and bottom positions (i.e., 90${}^{\circ }$ and 270${}^{\circ }$) and higher sensitivity for the results at 0${}^{\circ }$ and 180${}^{\circ }$ in absolute values.

Considering the polarity (or direction) of the sensors’ responses, the downward trend in the optical power variation (indicated by the negative sensitivity) occurs at the lateral section’s position of 0${}^{\circ }$ and 90${}^{\circ }$ as anticipated by the analytical results. In contrast, the opposite behavior occurs at 180${}^{\circ }$ and 270${}^{\circ }$ angular positions, as also anticipated by the analytical results. However, it is worth mentioning that the lower sensitivity obtained in angular position of 90${}^{\circ }$ can be due to additional stress components added in the POF when the lateral section was positioned at 90${}^{\circ }$, which can be related to the experimental setup conditions that resulted in higher torsion components or other transverse force elements not considered in the analytical model presented in Section 2.

Nevertheless, the sensitivity analysis at different cycles indicated high repeatability of the sensor, where similar sensitivities were obtained in all four cycles. Thus, such differences between the analytical and experimental results can be compensated by means of a sensor calibration at each lateral section’s angular position prior to its application in shape sensing.

Following the proposed methodology for shape reconstruction of the POF and multiplane angular position sensing, the angular velocity (if not already known in the application) can be estimated from a differential (or frequency) analysis of the sensor response, as shown in Figure 5. After knowing the angular velocity, the sensitivity of the sensor for angle variations (see Figure 7) can be used to estimate the *x*–*z* position of the optical fiber (or the lateral section position). Then, the last step is the estimation of the angular position at the *x*–*y* plane.

**Figure 8:**
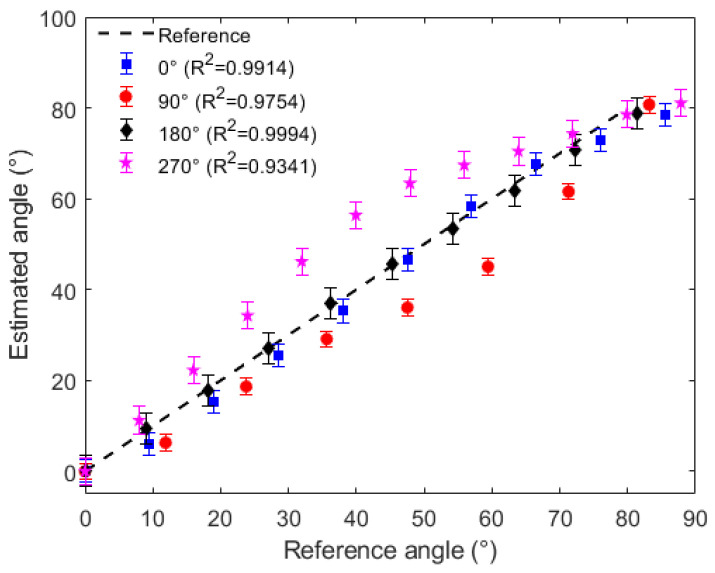
Angular position estimation at *x*–*y* plane for the POF sensor at different lateral section positions.

In this case, the sensor responses (as the ones presented in Figure 6) are normalized as a function of both the initial optical power and signal variation. Thereafter a linear regression is applied, where the optical power is converted into the angular position at the *x*–*y* plane. Figure 8 shows the estimated angles in the *x*–*y* plane for each lateral section angular position, where it is possible to observe a high correlation between the estimated and reference angles for all analyzed cases ($R^{2}$ higher than 0.9, which represents a strong correlation between the estimated and reference angles).

The RMSE value for the positions of 0${}^{\circ }$, 90${}^{\circ }$. 180${}^{\circ }$ and 270${}^{\circ }$ were 3.2363, 8.2134, 1.1523 and 9.5679, respectively. Thus, the results indicate the feasibility of using the proposed approach for the assessment of the angles at *x*–*z* and *x*–*y* planes as well as the influence of the different planes in the sensor response.

## 5. Conclusions

In this paper, we presented the application of POF-intensity-variation-based sensors for sensor shape reconstruction and multiplane angular positions assessment. A mathematical analytical model was applied using elasticity theory to simulate the stress and strain behavior on a loaded POF, and it was used to discuss if there exists a correlation between the sensor optical response and multiplanar forces/curvatures acting over it as well as to prove the feasibility of the proposed methodology of shape sensing. Cyclic curvature tests were performed in an experimental apparatus to characterize the sensor at different angular positions of the sensitive zone.

The results from the frequency responses demonstrated that the optical fiber sensors were useful for angular velocity estimation. The sensors presented no significant sensitivity variations at a specific lateral section position for different angular velocities of the test, which indicates good repeatability. The obtained coefficients of determination ($R^{2}$) for all calibration curves relative to the lateral section in different positions were higher than 0.9.

Thus, it is feasible to employ the proposed approach for multiplane angular position sensing and shape reconstruction considering that the angles estimated by the POF sensor had a high correlation with the reference angles measured by the potentiometer. It is intended that the approach may be a useful tool for shape reconstruction using POF sensors in different application scenarios, such as biomechanics, robotics and structural health monitoring.

Future works may include the viscoelastic behavior from the POFs on stresses and strains analysis in order to improve the mechanical bending model even if the one presented in this work was able to achieve a good correlation between the mechanical bending and the optical response of the POF sensor with the sensitive zone in different angular positions. Another work could be the application of the sensors in lower limb shape reconstruction during the human gait to assist in rehabilitation.

## Figures and Tables

**Table 1:** Polymer optical fiber dimensions and mechanical properties.

Parameter	Value
Fiber total radius (*R*)	1 mm
Lateral section depth (*h*)	0.81 mm
Fiber total length (*L*)	100 mm
Lateral section length (*C*)	20 mm
Fiber Young’s modulus (*E*)	3.09 GPa
Poisson ratio (υ)	0.3

## Data Availability

Data available by reasonable request.
